# Integration of Transcriptomics and Proteomics to Elucidate Inhibitory Effect and Mechanism of Antifungalmycin B from Marine *Streptomyces hiroshimensis* in Treating *Talaromyces marneffei*

**DOI:** 10.3390/md23020076

**Published:** 2025-02-10

**Authors:** Qiqi Li, Zhou Wang, Cuiping Jiang, Jianglin Yin, Yonghong Liu, Xinjian Qu, Xiangxi Yi, Chenghai Gao

**Affiliations:** 1 Institute of Marine Drugs/Faculty of Pharmacy, Guangxi University of Chinese Medicine, Nanning 530200, China; qiqi28208@163.com (Q.L.); wangzhou2312@163.com (Z.W.); ping990120@foxmail.com (C.J.); yingjianglin@126.com (J.Y.); yonghongliu@acsio.ac.cn (Y.L.); 2 Guangxi Key Laboratory of Marine Drugs/Guangxi University Engineering Research Center of High-Efficient Utilization of Marine Traditional Chinese Medicine Resources, Guangxi University of Chinese Medicine, Nanning 530200, China

**Keywords:** *Talaromyces marneffei*, antifungal mechanism, proteomics, transcriptomics

## Abstract

*Talaromyces marneffei* (TM) is an opportunistic pathogenic fungus that mainly infects immunocompromised patients. Currently, the global prevalence of talaromycosis caused by TM is increasing, leading to an increased demand for anti-TM drugs. In our previous study, a novel 28-membered macrolide compound, antifungalmycin B (ANB), was isolated from *Streptomyces hiroshimensis* GXIMD 06359, exhibiting significant antifungal properties. However, its in vivo mechanisms and direct antifungal effects warrant further investigation. In this study, we employed a mouse model in conjunction with transcriptomic and proteomic approaches to explore the antifungal activity of ANB against *T. marneffei*. In an in vivo mouse model infected with *T. marneffei* infection, ANB significantly reduced fungal burdens in the liver, spleen, lungs, and kidneys. Additionally, it markedly decreased the levels of reactive oxygen species (ROS) and cytokines, including interleukin (IL)-1β, IL-6, and tumor necrosis factor (TNF)-α. Proteomic and transcriptomic studies, complemented by parallel reaction monitoring (PRM) analysis, revealed that ANB effectively disrupted acid biosynthesis and cellular energy metabolism, thereby impairing mitochondrial functions in *T. marneffei*. These effects were exerted through multiple pathways. These findings highlight the potential of ANB as a versatile inhibitor of polyene macrolide-resistant fungi, offering a promising therapeutic avenue for the treatment of talaromycosis.

## 1. Introduction

*Talaromyces marneffei* infection leads to talaromycosis, a severe fungal disease predominantly found in the tropical and subtropical areas of Southeast Asia [1]. This invasive, systemic infection is especially dangerous for immunocompromised individuals who have a significantly high mortality rate, thus raising concerns [2]. The disease also poses a risk of becoming epidemic in new areas [3]. Since the mid-1990s, the prevalence of *T. marneffei* infection has increased not only among people living with human immunodeficiency virus (HIV) but also among those who are immunocompromised without HIV infection [4,5]. In a review published in 2018, *T. marneffei* was ranked as the third-most common fungal infection in the world. By 2021, experts from endemic regions advocated that it be officially recognized as a neglected tropical disease, stressing the global impact of this infection [6,7].

With the global rise in fungal infections, existing antifungal medications are inadequate, highlighting the critical need for new drugs to increase treatment effectiveness. Currently, three main classes of agents (polyenes, azoles, and echinocandins) are used to treat invasive talaromycosis. Amphotericin B (AmB) is the preferred initial treatment for severe cases of talaromycosis [8], but its high cost and severe side effects, including renal failure, electrolyte imbalances, vasculitis, and bone marrow suppression, limit its use [9]. Furthermore, azole antifungals have encountered resistance issues with *T. marneffei* [10,11]. Given these challenges, developing novel antifungal agents is an urgent and essential task to address the evolving threats of talatomycosis and other fungal diseases. Sangkanu et al. (2021) isolated 19 actinomycete extracts from marine organisms and sediments that showed antifungal activity against *T. marneffei* [12]. Additionally, Jiang et al. (2024) investigated a novel marine-derived compound called bamemacrolactine C, which has demonstrated antifungal activity against *T. marneffei* and is capable of disrupting the integrity of cell membranes and inhibiting intracellular enzyme activities [13]. Therefore, the search for new and efficient antibiotics from the marine sources with fewer side effects and lower costs is important for combating *T. marneffei.*

Antifungalmycin B (ANB), a novel polyene macrolide derived from the mangrove-associated *Streptomyces hiroshimensis* GXIMD 06359, has shown efficacy against *T. marneffei* [14,15]. Our preliminary study revealed that ANB acts on *T. marneffei* by compromising the integrity of its cell membrane and mitochondrial function, leading to apoptosis. These findings highlight the potential of ANB as an effective treatment for *T. marneffei* [14]. However, the specific mechanisms by which ANB disrupts mitochondrial function and damages cell membranes in *T. marneffei* remain underexplored. Although a few systematic studies exist, comprehensive studies on the intracellular responses of *T. marneffei* are lacking. The detailed antifungal mechanism of ANB in *T. marneffei* also remains unclear. Hence, further exploration is needed to investigate the potential antifungal effects of ANB in vivo in mouse models infected with *T. marneffei*.

Therefore, we investigated the antifungal activity of ANB in a mouse model of *T. marneffei* infection. To identify the intracellular targets affected by ANB, we employed both transcriptomic and proteomic approaches. Additionally, parallel reaction monitoring (PRM) was utilized to confirm changes in protein expression. This study helps to elucidate the mechanism of action and the in vivo efficacy of ANB against *T. marneffei* and provides valuable insights into the multitarget inhibitory mechanisms of polyene macrolides.

## 2. Results

### 2.1. Antifungalmycin B Demonstrated Significant Efficacy in an Immunocompromised Disseminated T. marneffei Model

**Figure 1:**
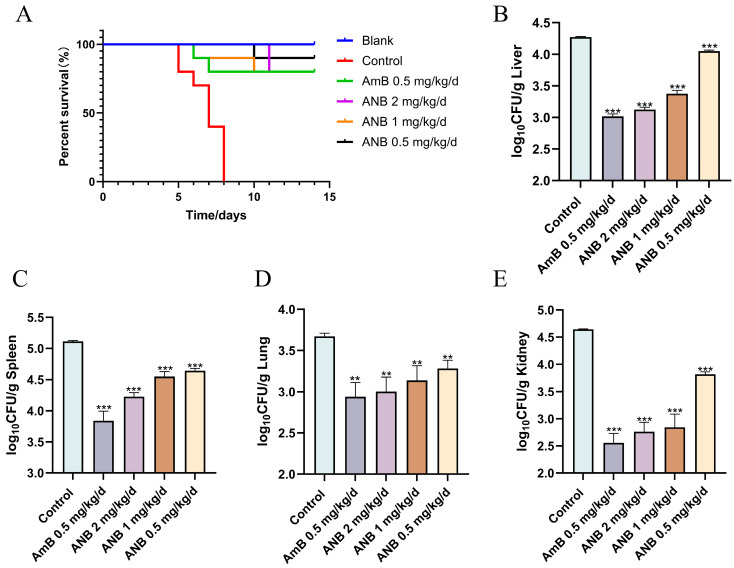
Therapeutic effect of ANB on talaromycosis in mice. (**A**) Survival curves for mice infected with *T. marneffei* and treated with ANB (administered intraperitoneally). (**B**–**E**) Quantitative analysis of fungal burden in tissues, expressed as colony-forming units (CFUs) per gram. Data are shown as mean ± S.D. with significance levels indicated as ** *p* < 0.01, and *** *p* < 0.001; statistical analysis performed using Student’s *t* test, n = 3.

To assess the in vivo antifungal effect of ANB, a murine model of immunocompromised disseminated *T. marneffei* was used. The mice were administered ANB, AmB, or saline for seven consecutive days. The results revealed that the mean survival time (MST) of the mice treated with saline was 7 days. In contrast, ANB affected the MST over 14 days. As depicted in Figure 1A, both ANB and AmB treatments significantly increased survival rates at day 14, with 80% survival at doses of 2 mg/kg/d or 1 mg/kg/d of ANB or 0.5 mg/kg/d AmB and 90% survival at 0.5 mg/kg/d) of ANB, while all the saline-treated mice died.

The severity of *T. marneffei* infection was assessed by determining the tissue fungal burden in mice from various experimental groups. As shown in Figure 1B–E, the fungal burden in the liver, spleen, lung, and kidney decreased after five days of daily intraperitoneal treatment with ANB. The quantification of fungal burden in the control group revealed that in the control group, the spleen was the most heavily affected organ, with a fungal burden of 5.11 ± 0.02 log10 CFU/g, followed by the kidney (4.65 ± 0.01 log10 CFU/g), liver (4.27 ± 0.01 log10 CFU/g), and lungs (3.67 ± 0.04 log10 CFU/g). Notably, a significant reduction in fungal burden was observed in the mice treated with 2 mg/kg/d, 1 mg/kg/d, and 0.5 mg/kg/d of ANB. The greatest decrease was observed in the group receiving 2 mg/kg/d of ANB compared with those treated with lower doses.

**Figure 2:**
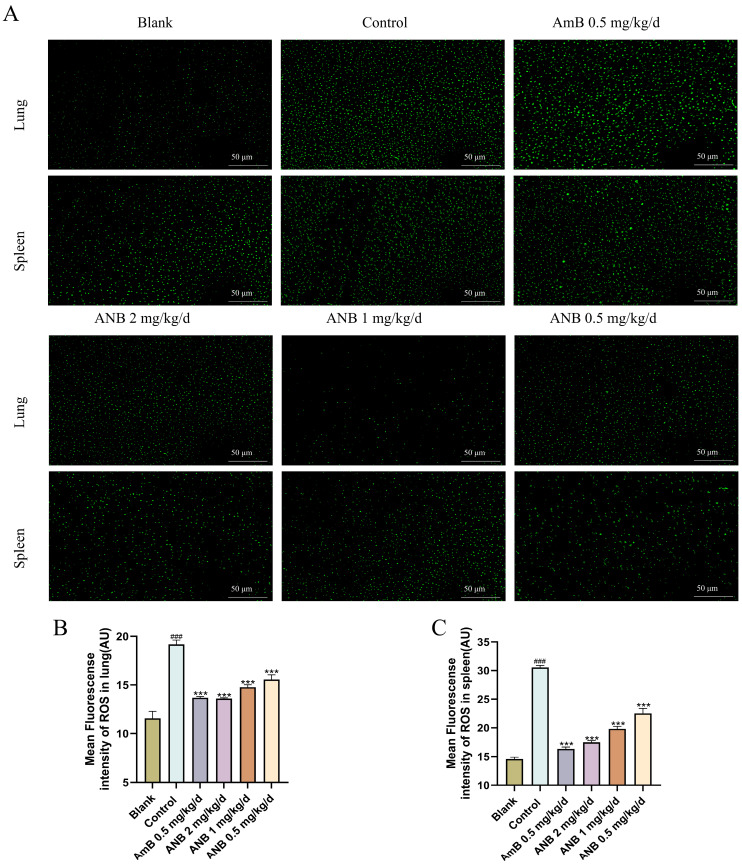
Measurement of ROS in cells of lung and spleen cells via an ROS fluorometric assay. (**A**) Fluorescence microscopy images of cells. (**B**,**C**) Quantitative evaluation of fungal burden in tissues, reported as CFUs per gram. The data are shown as means ± S.Ds. Control group vs Blank group: ^###^ *p* < 0.001. Treatment groups vs Control group: ***p < 0.001; Student’s *t* test, n = 3.

The intracellular levels of ROS in the spleen and lung were subsequently assessed (Figure 2). ROS are pivotal in regulating cell survival, with moderate levels supporting cell proliferation and survival. However, excessive ROS can induce oxidative stress, resulting in damage such as DNA strand breaks, protein degradation, lipid peroxidation, and ultimately cell death [16,17]. In the control group, the ROS levels significantly increased from 14.58 to 30.55 in the spleen and from 11.57 to 19.16 in the lungs (Figure 2B,C). In contrast, the level of ROS was significantly lower in the groups treated with ANB at doses of 2 mg/kg/d, 1 mg/kg/d, and 0.5 mg/kg/d. The cells in the spleen and lungs of the ANB-treated group presented weak green fluorescence, indicating reduced ROS accumulation, whereas the cells in the control group presented strong fluorescence, suggesting high ROS levels. These findings indicate that ANB can effectively reduce the accumulation of intracellular ROS in the spleen and lung.

**Figure 3:**
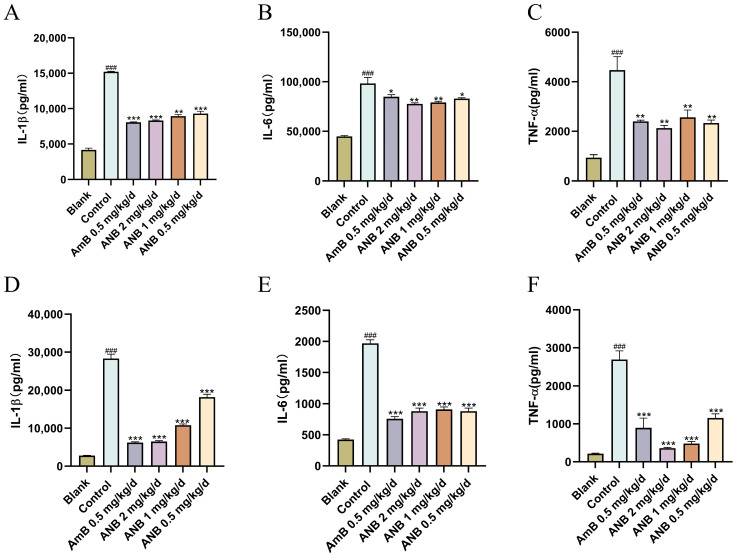
Anti-inflammatory activity of ANB in *T. marneffei*-infected mice. (**A**–**C**) Comparison of inflammatory factors in lung tissue. (**D**–**F**) Comparison of inflammatory factors in spleen tissue. The data are shown as means ± S.Ds. Control group vs Blank group: ^###^ *p* < 0.001. Treatment groups vs Control group: * *p* < 0.05, ** *p* < 0.01, and *** *p* < 0.001; Student’s *t* test, n = 3.

Cytokine levels were measured to assess whether the concentrations of inflammatory factors in challenged mice changed in the presence or absence of ANB. By day five of treatment, ANB had significantly lowered the levels of the proinflammatory cytokines IL-1β, IL-6, and TNF-α (Figure 3). These results indicate that ANB effectively controlled *T. marneffei* infection by decreasing the levels of proinflammatory IL-1β, IL-6, and TNF-α.

### 2.2. Transcriptomics of ANB-Treated T. marneffei

**Figure 4:**
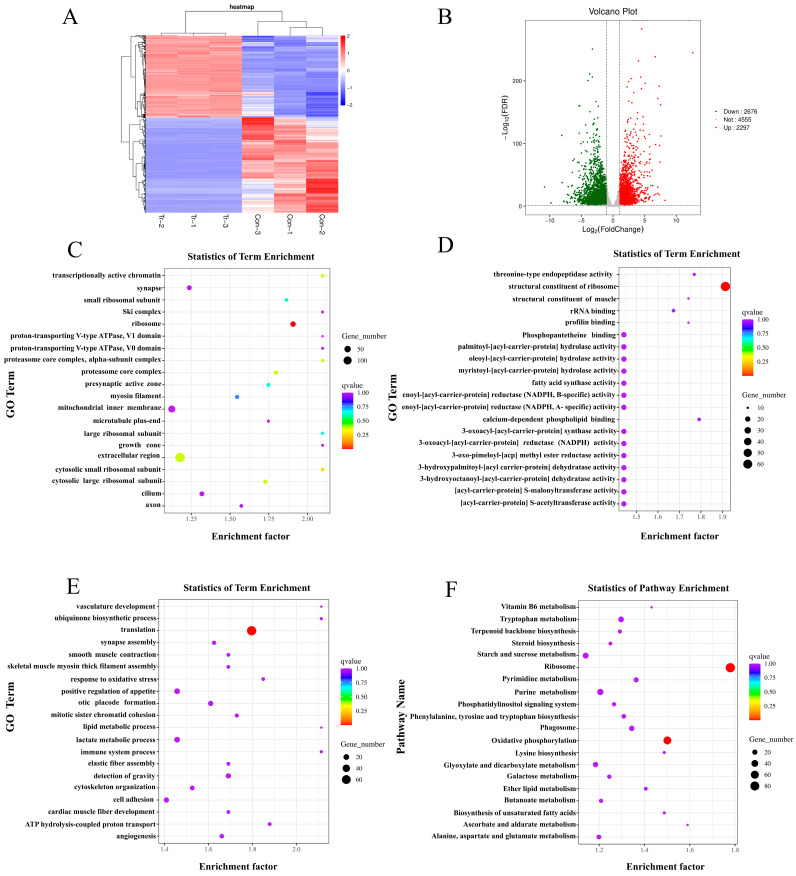
Transcriptomic alterations in *T. marneffei* following treatment with ANB. (**A**) Heatmap displaying differentially expressed genes (DEGs). Hierarchical clustering is shown, with changes in gene expression represented by the color scale. (**B**) Volcano plot illustrating DEGs; upregulated genes are highlighted in red, while downregulated genes are shown in green. (**C**) Top 20 cellular component terms from the Gene Ontology (GO) classification. (**D**) Top 20 molecular function terms from the GO classification. (**E**) Top 20 biological process terms from the GO classifications. (**F**) Top 20 pathways from the KEGG analysis.

The changes in the transcriptome of *T. marneffei* in response to ANB treatment were analyzed by RNA-Seq to obtain insights into the mechanism of action of ANB on *T. marneffei*. A total of 4973 genes were differentially expressed in the ANB-treated group, with 2297 genes being upregulated and 2676 genes being downregulated. A volcano plot provided a visual representation of the expression levels of each differentially expressed gene (Figure 4B). Hierarchical clustering analysis of the DEGs revealed consistent patterns within groups and distinct differences between groups, demonstrating the high reliability of the data (Figure 4A).

The DEPs were annotated in the GO database, resulting in a total of 1986 DEGs (40.90% of the total) being mapped to three GO categories: cellular component (CC), molecular function (MF), and biological process (BP). Within the CC category, the significantly enriched terms included “proton-transporting V-type ATPase, V1 domain”, “ proton-transporting V-type ATPase, V0 domain”, and “mitochondrial inner membrane” (Figure 4C). The MF category featured significantly enriched terms associated with lipid synthesis and biological metabolism, such as “fatty acid synthase activity”, “enoyl-[acyl-carrier-protein] reductase (NADPH, B-specific) activity”, “enoyl-[acyl-carrier-protein] reductase (NADPH, A-specific) activity”, and “3-oxoacyl-[acyl-carrier-protein] reductase (NADPH) activity” (Figure 4D). In the BP category, terms such as “lipid metabolic process”, “lactate metabolic process”, and “ATP hydrolysis coupled proton transport” were significantly represented (Figure 4E).

To gain a deeper understanding of the biological functions of DEGs, KEGG enrichment analysis was employed using a public database that provides information on various biochemical pathways. The analysis revealed that the DEGs were predominantly involved in pathways associated with biochemical metabolism and synthesis. Among the genes analyzed, 1732 DEGs (35.67%) were successfully mapped via KEGG enrichment analysis; the top 20 pathways are illustrated in Figure 4F. Notably, the pathways for “oxidative phosphorylation” and “biosynthesis of unsaturated fatty acids” were significantly enriched (Table S1 in the Supplementary Materials). These findings suggest that ANB can impact lipid synthesis and the overall biological metabolism in *T. marneffei*.

### 2.3. Proteomics of ANB-Treated T. marneffei

**Figure 5:**
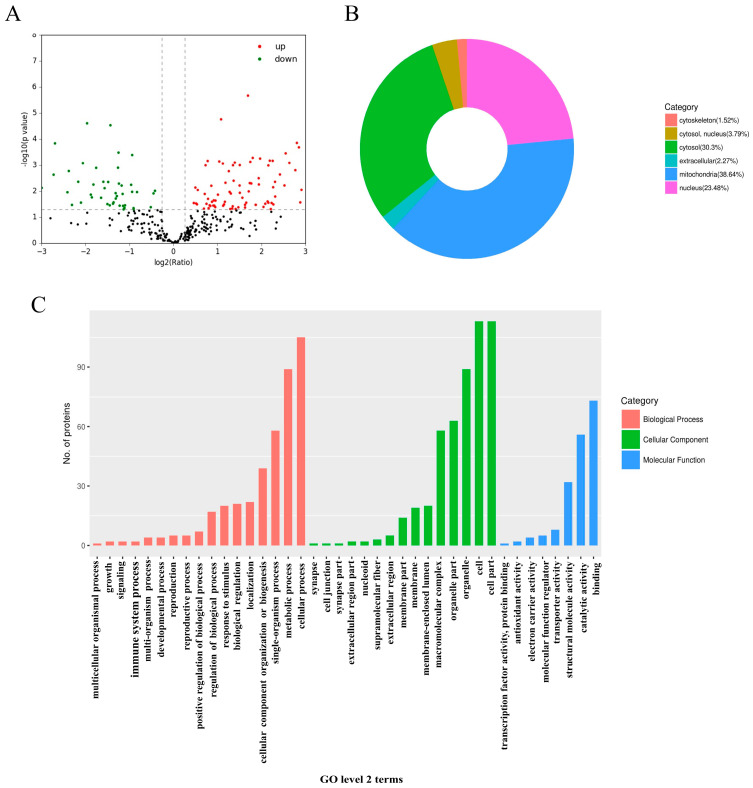
Proteome differences induced by ANB in *T. marneffei*. (**A**) Volcano plot displaying DEPs; proteins with increased expression are marked in red, while those with decreased expression are in green. (**B**) Distribution of subcellular localization terms for DEPs. (**C**) Level 2 statistics from the GO annotations. Analysis of correlations between transcriptomics and proteomics data.

Proteomic technology has emerged as a crucial tool for investigating the antifungal mechanisms of various compounds [18,19]. A total of 779 proteins were identified when *T. marneffei* was treated with ANB. The criteria for screening DEPs required that their expression levels be greater than 1.2-fold to be classified as upregulated, or less than 0.5-fold to be considered downregulated. Consequently, 132 DEPs were detected in the ANB-treated samples, with 85 proteins categorized as upregulated and 47 as downregulated (Figure 5A).

Subcellular location classification was used to gain further insight into the mechanism of action of ANB on *T. marneffei*, revealing that the DEGs were most enriched in the mitochondria domain (Figure 5B). To further dissect the functions of these DEGs, GO functional classification was applied, categorizing the DEPs according to BP, CC, and MF. As illustrated in Figure 5C, under the BP classification, the most represented subcategories of DEPs included cellular processes, metabolic processes, single-organism processes, and cellular component organization or biogenesis. For the CC classification, the leading subcategories were cell part, cell, organelle, and organelle part. In the MF classification, the top enriched subcategories were binding, catalytic activity, structural molecule activity, and transporter activity. During the treatment of *T. marneffei* with ANB, 20 DEPs were associated with the response to stimulation, 89 were associated with metabolic processes, 19 acted on the membrane surface, 14 were involved membrane parts, and 20 functioned within the membrane-enclosed lumen. These proteins may be involved in the mechanism of ANB against *T. marneffei* and play critical roles in fungal growth, development, pathogenicity, and metabolism.

**Figure 6:**
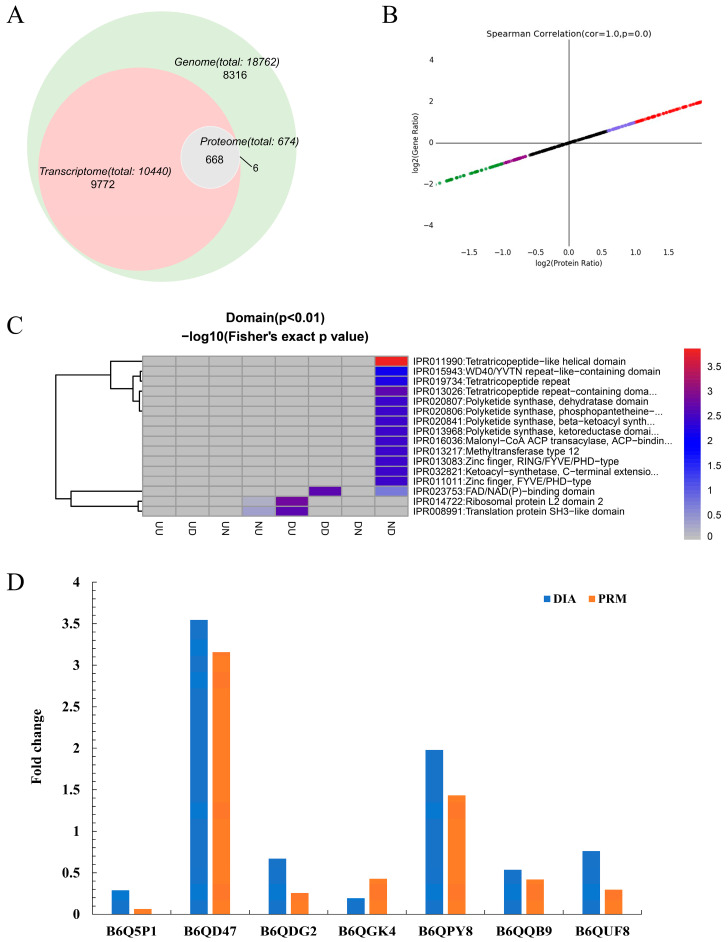
Joint transcriptomic and proteomic analysis of ANB in *T. marneffei*. (**A**) Venn diagram illustrating the overlap between the transcriptomic and proteomic data. (**B**) Correlation analysis between the transcriptome and proteome. (**C**) Analysis of domains within the transcriptome and proteome. (**D**) PRM analysis of 7 selected differentially expressed proteins.

Correlation analysis was employed to elucidate the relationships between the proteome and transcriptome. All genes and proteins involved in the association study are shown in Figure 6A. The findings, displayed in Figure 6B, revealed significant correlations among a total of 80 DEGs and DEPs from both groups, including forty-five “upregulated-upregulated”, six “upregulated-downregulated”, twenty-four “downregulated-upregulated”, and five “downregulated-downregulated”.

In a comprehensive examination combining both transcriptome and proteome data, domain analysis was prioritized. Genes and proteins were selected on the basis of their domains for a thorough combined analysis; the results are presented in Figure 6C. This integrated domain analysis revealed that DEPs and DEGs primarily characterized as “downregulated–downregulated” were significantly enriched in the FAD/NAD(P)-binding domain, indicating that ANB can effectively disrupt the biological metabolism of *T. marneffei*.

Additionally, detailed mappings of two closely associated metabolic pathways, the glycolytic pathway and the citrate cycle (TCA cycle), were performed. A total of 10 DEPs and 36 DEGs were identified as significant for these pathways (Tables S3 and S4 in Supplementary Materials). Both pathways exhibited a notable decreasing trend, with key enzymes such as pyruvate kinase (PK) and the α-oxoglutarate dehydrogenase complex (OGDHC) showing decreased expression levels; however, no corresponding genes were identified. Furthermore, seven proteins were linked to these pathways (B6Q5P1, B6QD47, B6QDG2, B6QGK4, B6QPY8, B6QQB9, and B6QUF8) were selected to test the accuracy of the proteomic data (Figure 6D). The results confirmed the proteomic findings and indicated that both the glycolytic pathway and the TCA cycle are inhibited in *T. marneffei* following ANB treatment.

## 3. Discussion

Owing to the limitations of current antifungal treatments such as azoles and AmB, new antifungal drugs for treating *T. marneffei* infection are urgently needed. Our previous study demonstrated that ANB significantly inhibits the growth and proliferation of *T. marneffei* in vitro by damaging cell membranes and impairing mitochondrial function [14]. Furthermore, in vivo studies confirmed the potent antifugal efficacy of ANB in a *T. marneffei* infection model. This study demonstrated that administering high concentrations of ANB significantly reduces fungal loads in vital organs of infected mice, including the liver, spleen, lungs, and kidneys, thereby aiding recovery and confirming the potential of ANB as a viable therapeutic option.

As byproducts of cellular metabolism, the ubiquitous presence of ROS indicates their fundamental role in biological systems [20]. While ROS play a supportive role in the immune system, they become cytotoxic when they accumulate excessively [21]. Excessive ROS can induce oxidative damage to DNA, proteins, and lipids within the body [20]. This study revealed a significant reduction in ROS levels following ANB treatment after severe *T. marneffei* infection, suggesting its potential to mitigate excessive ROS production. Importantly, previous studies have noted that hyperinflammatory responses lead to mortality in mice infected with *T. marneffei* [22]. Higher levels of proinflammatory cytokines were observed in patients with talaromycosis before treatment than in both HIV-infected individuals and healthy controls. These cytokine levels peaked three days after antifungal therapy and gradually declined. Correspondingly, the clinical symptoms of these patients also diminished over time [23]. Our findings indicate that ANB effectively reduced the concentrations of the proinflammatory cytokines IL-1β, IL-6, and TNF-α (Figure 3), likely due to the rapid clearance of *T. marneffei* from infected tissues. The literature supports the notion that compounds that reduce proinflammatory cytokines through pathogen inhibition can be effective. For example, Xiaoyun Liu et al. (2022) reported a significant reduction in TNF-α, IL-6, and IL-1β levels following SA-2 treatment in intranasal *C. neoformans* infection, which aligns with our findings [24]. Additionally, eugenol treatment was shown to decrease the levels of proinflammatory cytokines (IL-1β, TNF-α, and iNOS) in *Aspergillus fumigatus*-infected mouse corneas [25]. Collectively, these studies suggest that successful antifungal therapy may alleviate inflammatory responses.

Energy metabolism, carbohydrate metabolism, and lipid metabolism serve as critical energy sources in fungal cells. DEG annotation via the KEGG pathway analysis revealed that ANB treatment substantially affected the energy metabolism and lipid synthesis pathways of *T. marneffei*. Furthermore, KEGG pathway enrichment analysis highlighted the substantial effects of ANB on oxidative phosphorylation and the biosynthesis of unsaturated fatty acids, both of which are essential for cellular metabolism. The plasma membrane protein PMA1, a plasma membrane H^+^-ATPase, plays a pivotal role in proton transport within the oxidative phosphorylation pathway. This protein belongs to a family of ATPases embedded in the cell membrane and is responsible for ion transport across cellular membranes powered by transient ATP phosphorylation [26,27]. The significance of PMA1 as a membrane protein has been demonstrated through various methods, including the disruption of the *PMA1* gene, RNA interference studies [28], and loss-of-function mutations in yeast [29]. Extensive investigations have also explored the potential of targeting PMA1 as an antifungal strategy across multiple fungal species. Gene silencing of *PMA1* leads to reduced cell growth and pathogenicity in *Penicillum digitatum* [30], whereas PGA treatment significantly decreases PMA1 activity, reducing stress tolerance in *Fusarium solani* [31]. These findings align with the results of our current study, where ANB treatment significantly downregulated PMA1 gene expression, thereby impairing energy metabolism in *T. marneffei*. Fatty acid synthase (FAS), an enzyme crucial for the biosynthesis of endogenous fatty acids [32], has a unique α6β6 configuration in fungi, with its seven-component activities distributed across two nonidentical polypeptides, α and β [33]. The notable structural differences between fungal and mammalian cells render this enzyme a promising target for the novel antifungal drugs [34]. The latest evidence indicates that the enzyme FAS holds significant potential as a target for antifungal drug development. Cerulenin forms a covalent bond with the catalytic site of FAS, thereby effectively inhibiting the biosynthesis of fatty acids and sterols in yeast [35]. The antifungal effects of trans-chalcone and quercetin on *Trichophyton rubrum* have been demonstrated by their ability to reduce ergosterol levels and modulate *FAS1/2* expression [36]. In this study, treatment with ANB resulted in a decreased expression of FAS1/2 in *T. marneffei* (Table S2 in Supplementary Materials). The transcriptomic analyses suggest that ANB targets *FAS1/2,* leading to the inhibition of essential fatty acid biosynthesis, findings that are consistent with those reported by Kali R. Iyer et al. [37].

The glycolysis pathway is ubiquitous across organisms and acts as a critical energy source for various biological activities; it is one of the fundamental metabolic pathways in microorganisms, providing the primary energy required for their biological functions [38]. PK plays a pivotal role by directly generating ATP and acts as the rate-limiting enzyme within the glycolytic pathway [39]. In this study, we demonstrated that ANB treatment significantly reduces PK activity in *T. marneffei*, leading to decreased pyruvate accumulation. A reduction in pyruvate disrupts the normal function of the TCA cycle, a central energy-producing pathway in eukaryotic cells that is critical for aerobic respiration in all life forms.

The OGDHC enzyme complex, which is essential for the oxidative decarboxylation of α-oxoglutarate within the TCA cycle, plays a crucial role in energy production, energy metabolism, and amino acid metabolism [40]; it has been shown to be involved in the energy metabolism of *Verticillium dahlia* and is critical for full virulence because of its ability to regulate various fungal developmental processes [41]. Our findings demonstrate that ANB treatment significantly reduced OGDHC activity in *T. marneffei*, thereby impairing the TCA cycle, as evidenced by decreased OGDHC activity and reduced pyruvate levels. Consistent with previous studies, ANB has been shown to markedly lower intracellular ATP levels and diminish the activities of enzymes such as malate dehydrogenase (MDH) and succinate dehydrogenase (SDH), leading to mitochondrial dysfunction and energy metabolism disruption in *T. marneffei* [14]. On the basis of these observations, we hypothesize that ANB treatment may reduce the activity of key TCA cycle enzymes and inhibit pyruvate accumulation by affecting PK activity, thereby suppressing energy metabolism in *T. marneffei*.

**Figure 7:**
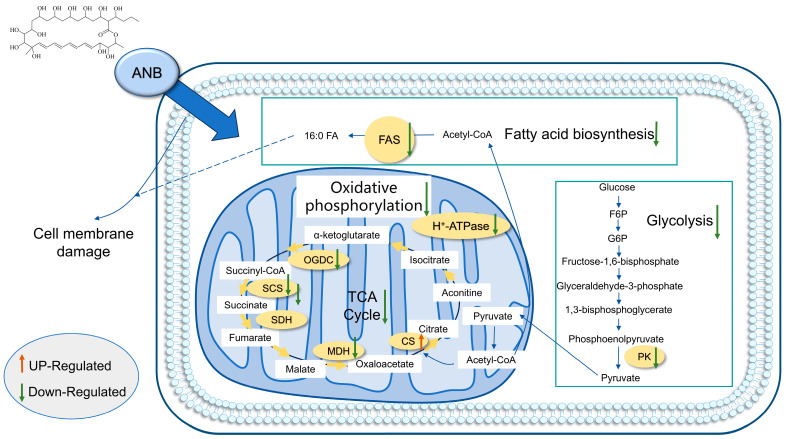
Hypothetical diagram of the mechanism of action of ANB against *T. marneffei*.

In this study, we constructed a hypothetical model elucidating the inhibitory mechanism of ANB against *T. marneffei* through an integrative analysis of proteomics and transcriptomics data from ANB-treated *T. marneffei* in conjunction with our previous investigations (Figure 7). ANB exerts its inhibitory effect by downregulating the expression of genes and proteins involved in fatty acid biosynthesis, mitochondrial function, and the respiratory chain. This downregulation results in mitochondrial dysfunction and triggers an oxidative stress response. The interruption of these critical metabolic processes ultimately results in cell death. This model offers valuable insights into the potential antifungal mechanism of ANB against *T. marneffei*.

In summary, we conducted an in vivo evaluation of the antifungal activity of ANB against *T. marneffei*. Our findings demonstrated that ANB significantly reduced the fungal burden and effectively mitigated the levels of ROS and proinflammatory cytokines in a murine model of *T. marneffei* infection. Through comprehensive proteomic and transcriptomic analyses, we revealed that ANB likely impairs the growth and virulence of *T. marneffei* by disrupting pathways associated with fatty acid biosynthesis and energy metabolism. Although additional experiments are needed to fully elucidate the underlying mechanisms, the extensive testing conducted in this research highlights the potential of ANB as a promising therapeutic option for *T. marneffei*. To validate the accuracy of ANB for the inhibition of the *T. marneffei* pathway, we will further detect the expression levels of key proteins of the signaling pathway, such as pyruvate kinase and α-oxoglutarate dehydrogenase, and changes in the concentrations of related metabolites. This research highlights not only the direct antifungal activity of ANB but also its role in modulating host immune responses, which may be critical for developing more effective therapeutic strategies against fungal infections.

## 4. Materials and Methods

### 4.1. Experimental Strains and Animals

The reference strain (*Talaromyces marneffei* ATCC 58950) was provided by Prof. YE Li from Guangxi Key Laboratory of AIDS Prevention and Control, Guangxi Medical University. For all experiments, we used a seven-day-old pure yeast culture grown on brain–heart infusion (BHI) agar. The *T. marneffei* colonies were thoroughly washed three times with phosphate-buffered saline (PBS) and then counted using a hemocytometer. The cells were resuspended in PBS and subjected to vigorous vortexing.

Male Institute of Cancer Research (ICR) mice, aged six to eight weeks and weighing between 18 and 22 g, were obtained from Hunan SJA Laboratory Animal Co., Ltd (Hunan, China). These mice were accompanied by an animal quality certificate that verified their specific pathogen free (SPF) status under license number SCXK 2019-0004.

### 4.2. Drugs

ANB was isolated from the fermentation broth of the mangrove-derived strain *Streptomyces hiroshimensis* GXIMD 06359 [14,15]. This compound was stored at the Institute of Marine Drugs, Guangxi University of Chinese Medicine. Cyclophosphamide and AmB were both provided by Shanghai Macklin Biochemical Co., Ltd (Shanghai, China).

### 4.3. Antifungal Activity In Vivo, with the Exception of the Control Group

Male ICR mice aged 6 to 8 weeks and weighing between 18 and 22 g were obtained from SPF Biotechnology. The mice were kept in standard cages with free access to water and food and maintained at a temperature of 21 to 23 °C with proper lighting. The mice were acclimatized for one week prior to the start of the experiments. Before *T. marneffei* inoculation, the mice were subjected to immunosuppression by a single intraperitoneal (i.p.) injection of cyclophosphamide at a dosage of 200 mg/kg per day for 4 days. To induce systemic infection, this yeast suspension was injected intraperitoneally into each mouse. The mice were randomly divided into five groups (n = 20). The experimental groups received an intraperitoneal injection of ANB at doses of either 2 mg/kg, 1 mg/kg, or 0.5 mg/kg. The control group was administered the same volume of normal saline or AmB at a concentration of 0.5 mg/kg (0.2 mL per mouse). The treatment was initiated 2 h post-inoculation and continued for a period of 7 days.

For survival analysis, 10 mice were assigned to each group and monitored daily until day 14 after inoculation. The analysis of the tissue fungal load involved sacrificing five mice from each group at 5 days post-inoculation. The livers, lungs, spleens, and kidneys were removed aseptically, weighed, and homogenized in 1 mL of sterile saline solution. The homogenates were then serially diluted in tenfold increments and plated on Sabouraud dextrose agar (SDA), followed by incubation at 37 °C for 72 h. After incubation, the number of colony-forming units (CFUs) per gram of tissue was determined. The levels of reactive oxygen species (ROS) were measured using a ROS fluorometric assay kit (Elabscience, Wuhan, China). Additionally, the levels of interleukin (IL)-1β, interleukin (IL)-6, and tumor necrosis factor (TNF)-α were quantified using a mouse ELISA kit (Elabscience, Wuhan, China) according to the manufacturer’s protocol.

### 4.4. Transcriptomic Analysis

The initial inoculum of *T. marneffei* cells was adjusted to a 1–5 × 10^4^ CFU mL^−1^ standard in 20 mL of RPMI-1640 medium and cultured for 72 h at 37 °C with shaking at 180 rpm. The treatment group received ANB at a concentration of 8 μg/mL. The concentration of 8 μg/mL is equal to the ANB 1/2MIC for *T. marneffei*. The cells were harvested, washed three times with sterile PBS, and then rapidly frozen in liquid nitrogen before being stored at −80 °C until further use. The cells from the untreated *T. marneffei* group served as the control. Three separate cultures were prepared for RNA-Seq analysis. The transcriptomic analysis was conducted by MHelix BioTech Co., Ltd. (Shanghai, China), involving steps such as total RNA extraction, mRNA enrichment by oligo (dT), RNA fragmentation, cDNA synthesis initiated by random hexamers, size selection, polymerase chain reaction amplification, and sequencing using an Illumina HiSeq 2500 platform.

The paired-end clean reads were aligned to the *T. marneffei* strain reference genome (https://www.ncbi.nlm.nih.gov/datasets/genome/GCF_009556855.1/, accessed on 8 January 2024) using HISAT2.2.4. To normalize gene expression across samples, the number of fragments per kilobase of transcript per million mapped reads value was calculated. Differentially expressed genes (DEGs) between the allicin-exposed and control groups were identified using DESeq2 on the basis of a false discovery rate (FDR) of less than 0.05 and an absolute fold change (FC) of greater than 1.

To explore the biological functions of the DEGs, Gene Ontology (GO) enrichment analysis was conducted using the GO database (http://geneontology.org/, accessed on 8 January 2024), and Kyoto Encyclopedia of Genes and Genomes (KEGG) pathway enrichment analysis was carried out using the KEGG database (https://www.genome.jp/kegg/, accessed on 8 January 2024).

### 4.5. Proteomics and PRM Analysis

The initial inoculum of *T. marneffei* cells was standardized to a concentration of 1–5 × 10^4^ CFU /mL in 20 mL of RPMI-1640 medium and incubated for 72 h at 37 °C with continuous shaking at 180 rpm. The treated group was then exposed to ANB at a final concentration of 8 μg/mL. The cells were then harvested and washed three times with sterile PBS. Before being rapidly frozen in liquid nitrogen, the samples were stored at −80 °C until analysis. The cells from the untreated group served as the control. Three independent cultures were prepared for RNA-Seq analysis, and triplicate samples from these cultures were submitted to MHelix BioTech Co., Ltd. (Shanghai, China), for TMT-based quantitative proteomic analysis.

The differentially expressed proteins (DEPs) identified in *T. marneffei* mycelia were defined as those showing a minimum 1.20-fold change and a *p*_value less than 0.05 when the ANB-treated samples were compared with the control samples. For GO annotation and KEGG enrichment analysis of DEPs, the web-based analysis tool Omicsbean (http://www.omicsbean.cn, accessed on 25 December 2023) was used.

The samples employed for omics PRM verification were identical to those used in the proteomic analysis. MHelix BioTech Co., Ltd. (Shanghai, China), was entrusted with performed PRM verification on 7 target proteins.

### 4.6. Statistical Analysis

The statistical analyses were carried out using GraphPad Prism 8.3.0 (GraphPad Software, San Diego, CA, USA), and the results are expressed as means and standard deviations (SDs). Each experiment was conducted with independent biological replicates. The data were analyzed using either one-way ANOVA or an unpaired *t*_test depending on the experimental design. Statistical significance was considered probability (*p*) values of less than 0.05, 0.01, or 0.001.

## Data Availability

The data presented in this study are available upon request from the corresponding author.

## Supplementary Materials

The following supporting information can be downloaded at: https://www.mdpi.com/article/10.3390/md23020076/s1, Table S1: Differential genes related to oxidative phosphorylation pathway. Table S2: Differential genes related to fatty acid biosynthesis pathway. Table S3: Differentially expressed genes and proteins associated with glycolysis. Table S4: Tricarboxylic acid cycle related differentially expressed genes and proteins.
