# Supplementary material for: Integration of Transcriptomics and Proteomics to Elucidate Inhibitory Effect and Mechanism of Antifungalmycin B from Marine Streptomyces hiroshimensis in Treating Talaromyces marneffei
Source: Mar Drugs. 2025 Feb 10;23(2):76. doi: 10.3390/md23020076 (PMC11857274; doi:10.3390/md23020076)
Supplement: Supplementary file 1 [file marinedrugs-23-00076-s001.zip › marinedrugs-3430955-supplementary.pdf]

Supplementary Information

Integration of transcriptomics and proteomics to elucidate inhibitory effect and mechanism of Antifungalmycin B from marine *Streptomyces hiroshimensis* in treating *Talaromyces marneffei*

Qiqi Li. *et al.*

List of Supporting Information

Table S1. Differential genes related to oxidative phosphorylation pathway.

Table S2. Differential genes related to fatty acid biosynthesis pathway.

Table S3. Diferentially expressed genes and proteins associated with glycolysis.

TableS4. Tricarboxylic acid cycle related dffrentially expressed genes and proteins.

Table S1. Differential genes related to oxidative phosphorylation pathway.

Sequence	Description	Gene ID	Gene_ FC
1	COX5B; cytochrome c oxidase subunit 5b	EYB26_008124	3.08224072
2	COX6A; cytochrome c oxidase subunit 6a	EYB26_008905	2.262598176
3	NDUFA9; NADH dehydrogenase (ubiquinone) 1 alpha subcomplex subunit 9	EYB26_003901	1.612019156
4	ATPeV1C; V-type H+-transporting ATPase subunit C	EYB26_005467	1.661657246
5	NDUFB3; NADH dehydrogenase (ubiquinone) 1 beta subcomplex subunit 3	EYB26_002228	1.308807591
6	NDUFA6; NADH dehydrogenase (ubiquinone) 1 alpha subcomplex subunit 6	EYB26_009701	1.287439753
7	ATPeV1B; V-type H+-transporting ATPase subunit B	EYB26_006240	1.150602238
8	ATPeV1E; V-type H+-transporting ATPase subunit E	EYB26_000329	1.807928397

9	SDHA; succinate dehydrogenase (ubiquinone) flavoprotein subunit	EYB26_004355	1.806831087
10	NDUFB8; NADH dehydrogenase (ubiquinone) 1 beta subcomplex subunit 8	EYB26_009588	3.116852792
11	ATPeV0C; V-type H+-transporting ATPase 16kDa proteolipid subunit	EYB26_009276	1.130928366
12	NDUFA5; NADH dehydrogenase (ubiquinone) 1 alpha subcomplex subunit 5	EYB26_003534	2.44977619
13	COX15; cytochrome c oxidase assembly protein subunit 15	EYB26_001685	2.589845593
14	QCR7; ubiquinol-cytochrome c reductase subunit 7	EYB26_001262	1.260015214
15	UQCRFS1; ubiquinol-cytochrome c reductase iron-sulfur subunit	EYB26_005972	1.172612672
16	NDUFV2; NADH dehydrogenase	EYB26_001365	2.055303602

	(ubiquinone) flavoprotein 2		
17	NDUFA8; NADH dehydrogenase (ubiquinone) 1 alpha subcomplex subunit 8	EYB26_006738	1.464788918
18	ATPeF1B; F-type H+-transporting ATPase subunit beta	EYB26_006715	1.200165774
19	COX4; cytochrome c oxidase subunit 4	EYB26_006716	2.367776909
20	COX17; cytochrome c oxidase assembly protein subunit 17	EYB26_004826	1.076732069
21	ATPeFG; F-type H+-transporting ATPase subunit g	EYB26_002806	1.383070652
22	ATPeV0E; V-type H+-transporting ATPase subunit e	EYB26_003590	1.657362986
23	ATPeV0C; V-type H+-transporting ATPase 16kDa proteolipid subunit	EYB26_004024	1.022543187
24	QCR8; ubiquinol-cytochrome c reductase subunit 8	EYB26_009990	1.42680194

25	COX7C; cytochrome c oxidase subunit 7c	EYB26_001172	2.053901709
26	ndh; NADH dehydrogenase	EYB26_002689	1.712287034
27	PMA1; H+-transporting ATPase	EYB26_007018	-3.333448711
28	ATPeF0C; F-type H+-transporting ATPase subunit c	EYB26_007962	1.437932956
29	NDUFS2; NADH dehydrogenase (ubiquinone) Fe-S protein 2	EYB26_001977	1.671584533
30	QCR6; ubiquinol-cytochrome c reductase subunit 6	EYB26_005358	2.609036977
31	ATPeV1A; V-type H+-transporting ATPase subunit A	EYB26_004172	1.297651401
32	NDUFV1; NADH dehydrogenase (ubiquinone) flavoprotein 1	EYB26_005326	2.394030718
33	SDHA; succinate dehydrogenase (ubiquinone) flavoprotein subunit	EYB26_009824	1.350863574

34	COX5A; cytochrome c oxidase subunit 5a	EYB26_004132	2.203499808
35	NDUFS7; NADH dehydrogenase (ubiquinone) Fe-S protein 7	EYB26_001293	1.771755492
36	ATPeV1H; V-type H+-transporting ATPase subunit H	EYB26_005452	1.469428354
37	NDUFB7; NADH dehydrogenase (ubiquinone) 1 beta subcomplex subunit 7	EYB26_000359	2.445641372
38	ATPeFH; F-type H+-transporting ATPase subunit h	EYB26_000168	1.251497321
39	ATPeV0B; V-type H+-transporting ATPase 21kDa proteolipid subunit	EYB26_002147	1.124455849
40	COX11; cytochrome c oxidase assembly protein subunit 11	EYB26_007312	1.83976441
41	COX6B; cytochrome c oxidase subunit 6b	EYB26_006277	2.487947703
42	ATPeF1D; F-type H+-transporting ATPase	EYB26_008572	1.280303553

	subunit delta		
43	TIM11; F-type H+-transporting ATP synthase subunit e	EYB26_007711	1.029740051
41	COX6B; cytochrome c oxidase subunit 6b	EYB26_006277	2.487947703
42	ATPeF1D; F-type H+-transporting ATPase subunit delta	EYB26_008572	1.280303553
43	TIM11; F-type H+-transporting ATP synthase subunit e	EYB26_007711	1.029740051
44	NDUFS6; NADH dehydrogenase (ubiquinone) Fe-S protein 6	EYB26_004190	1.577616073
45	SDHD; succinate dehydrogenase (ubiquinone) membrane anchor subunit	EYB26_002738	2.489689474
46	QCR9; ubiquinol-cytochrome c reductase subunit 9	EYB26_005945	1.138518628
47	SDHC; succinate dehydrogenase (ubiquinone) cytochrome b560 subunit	EYB26_003674	5.771029607

48	CYC1; ubiquinol-cytochrome c reductase cytochrome c1 subunit	EYB26_004932	1.621556471
49	ATPeV1F; V-type H+-transporting ATPase subunit F	EYB26_003583	1.331117006
50	PMA1; H+-transporting ATPase	EYB26_004307	-3.904182734
51	NDUFS5; NADH dehydrogenase (ubiquinone) Fe-S protein 5	EYB26_000896	2.597125079
52	NDUFS4; NADH dehydrogenase (ubiquinone) Fe-S protein 4	EYB26_005814	1.891075924
53	ATPeFF; F-type H+-transporting ATPase subunit f	EYB26_005813	1.342083867
54	ATPeV0D; V-type H+-transporting ATPase subunit d	EYB26_000424	1.282706174
55	NDUFA2; NADH dehydrogenase (ubiquinone) 1 alpha subcomplex subunit 2	EYB26_000193	1.327878474
56	ATPeFK; F-type H+-	EYB26_004574	1.144127406

	transporting ATPase subunit k		
57	ppa; inorganic pyrophosphatase	EYB26_009816	1.40484405
58	NDUFS3; NADH dehydrogenase (ubiquinone) Fe-S protein 3	EYB26_002632	1.574430102

Table S2. Differential genes related to fatty acid biosynthesis pathway.

Sequence	Description	Gene ID	Gene_ FC
1	fabG; 3-oxoacyl-[acyl-carrier protein] reductase	EYB26_007596	-1.214559979
2	FAS2; fatty acid synthase subunit alpha, fungi type	EYB26_007410	-1.48387266
3	FAS1; fatty acid synthase subunit beta, fungi type	EYB26_005505	-2.212732207
4	fabF; 3-oxoacyl-[acyl-carrier-protein] synthase II	EYB26_005784	-2.163709367
5	MECR; mitochondrial enoyl-[acyl-carrier protein] reductase / trans-2-enoyl-CoA reductase	EYB26_009154	1.878104867
6	FAS1; fatty acid synthase subunit beta, fungi type	EYB26_007408	-2.971329171
7	fabD; [acyl-carrier-protein] S-malonyltransferase	EYB26_001295	1.191037026
8	fabG; 3-oxoacyl-[acyl-carrier protein] reductase	EYB26_003029	3.334812692
9	ACSL; long-chain acyl-CoA synthetase	EYB26_007004	-1.306451119
10	fabG; 3-oxoacyl- [acyl-carrier protein] reductase	EYB26_004247	7.290608832

Table S3. Diferentially expressed genes and proteins associated with glycolysis.

Sequence	Description	Gene ID	Gene_ FC	Protein ID	Protein _ FC
1	Glucose-6-phosphate isomerase			B6QD47	3.1572
2	phosphoglycerate mutase (2,3-diphosphoglycerate-independent)			B6QQZ2	2.02026
3	Dihydrolipoyl dehydrogenase			B6QDG2	0.25669
4	Pyruvate kinase			B6QLL0	0.22326
5	Glyceraldehyde-3-phosphate dehydrogenase	EYB26_005170	1.846541054	B6QPY8	1.43226
6	Pyruvate decarboxylase	EYB26_000781、EYB26_002404	2.922035078、-2.068083368	B6Q5P1	0.06452
7	Alcohol dehydrogenase,	EYB26_008794、	-3.942344696	B6QUF8	0.29618

	putative	EYB26_009405	` 4.139296221
8	phosphoglucomutase PgmA	EYB26_000179	1.707177
9	aldose 1-epimerase, putative	EYB26_000408	1.134358
10	oxidoreductase, zinc-binding	EYB26_000845	-2.041727056
11	hexokinase Kxk, putative	EYB26_001345	3.365587156
12	phosphoenolpyruvate carboxykinase AcuF	EYB26_001690	-3.204783187
13	triosephosphate isomerase	EYB26_001873	1.078262079
14	hypothetical protein	EYB26_002600	-2.13552
15	glucokinase, putative	EYB26_002848	-3.24747
16	hypothetical protein	EYB26_003128	1.12695
17	hypothetical	EYB26_003829	-2.39076

	protein		
18	phosphoglucomutase, putative	EYB26_004807	1.069974
19	hypothetical protein	EYB26_004953	-1.76248
20	acetate--CoA ligase	EYB26_005323	1.663043
21	Aldehyde dehydrogenase	EYB26_006542	-3.6127
22	possible apospory-associated protein c	EYB26_008017	1.305996
23	aldehyde dehydrogenase, putative	EYB26_008367	-2.71348
24	aldehyde dehydrogenase, putative	EYB26_008793	-3.88531
25	aldose 1-epimerase, putative	EYB26_009058	2.67812
26	pyruvate dehydrogenase E1 beta subunit PdbA, putative	EYB26_009152	1.179216

Table S4. Tricarboxylic acid cycle related dffrentially expressed genes and proteins.

Sequence	Description	Gene ID	Gene_ FC	Protein ID	Protein_ FC
1	Citrate synthase	EYB26_007011	1.426640237	B6QHT1	1.687
2	Succinate--CoA ligase [ADP-forming] subunit beta, mitochondrial			B6QGK4	0.42886
3	Dihydrolipoyl dehydrogenase			B6QDG2	0.25669
4	oxoglutarate dehydrogenase (succinyl-transferring)			B6QQB9	0.42133
5	fumarate hydratase, putative	EYB26_000291	1.720929598		
6	phosphoenolpyruvate carboxykinase AcuF	EYB26_001690	-3.204783187		
7	hypothetical protein	EYB26_002041	1.220148119		
8	ATP citrate lyase subunit (Acl), putative	EYB26_002042	2.365916525		
9	hypothetical protein	EYB26_002600	-2.1355223		
10	succinate dehydrogenase	EYB26_002738	2.489689474		

	subunit CybS, putative	
11	isocitrate dehydrogenase, NAD-dependent	EYB26_002971 1.36606609
12	NAD(+)-isocitrate dehydrogenase subunit I	EYB26_0033662.284234975
13	hypothetical protein	EYB26_0036745.771029607
14	hypothetical protein	EYB26_0043551.806831087
15	hypothetical protein	EYB26_0054691.491683985
16	pyruvate carboxylase, putative	EYB26_0071202.777246306
17	pyruvate dehydrogenase E1 beta subunit PdbA, putative	EYB26_0091521.179215939
18	succinate dehydrogenase subunit Sdh1, putative	EYB26_0098241.350863574
